# Patient-specific modeling of G2/M cell cycle dynamics reveals prognostic dynamical regimes driven by copy number alterations

**DOI:** 10.1038/s41540-026-00766-4

**Published:** 2026-06-08

**Authors:** Andrea Tonina, Alessandro Romanel

**Affiliations:** Department of Cellular, Computational and Integrative Biology (CIBIO), University of Trento, Trento, Italy

**Keywords:** Cancer, Computational biology and bioinformatics, Systems biology

## Abstract

Cell cycle dysregulation is a hallmark of cancer and a major source of tumor heterogeneity. While large-scale cancer genomics studies have characterized recurrent copy number alterations affecting cell cycle regulators, how these alterations translate into patient-specific dynamical dysregulation remains poorly understood. Here, we develop a stochastic, human-specific model of the G2/M cell cycle transition and integrate somatic copy number alterations to generate patient-specific perturbations of cell cycle dynamics. Copy number profiles from breast cancer patients are quantitatively mapped to model components, enabling simulation of individualized G2/M transition behavior. Model simulations reveal that copy number alterations induce distinct dynamical regimes characterized by systematic shifts in transition timing and switching commitment. Importantly, model-derived dynamical metrics stratify patients into groups with significantly different overall survival, independently of age and molecular subtype. Patients exhibiting delayed or impaired G2/M transitions show improved survival probability, consistent with reduced proliferative capacity. These results demonstrate that mechanistic, stochastic modeling can transform static genomic alterations into clinically relevant dynamical phenotypes, highlighting the potential of systems-level approaches to bridge cancer genomics and functional tumor behavior.

## Introduction

Uncontrolled cell proliferation is a defining hallmark of cancer and arises from dysregulation of the cell cycle control system^1,2^. Alterations affecting cyclin-dependent kinases, cyclins, phosphatases, and checkpoint regulators are pervasive across tumor types and contribute to genomic instability and malignant progression^3^. Among cell cycle checkpoints, the G2/M transition plays a critical role by ensuring that cells enter mitosis only after successful DNA replication and repair, and its dysregulation has been widely implicated in cancer development^4–6^.

Large-scale cancer genomics studies have generated comprehensive catalogs of somatic alterations affecting cell cycle regulators, including recurrent copy number alterations (CNAs) that modulate gene dosage^7,8^. However, while these datasets provide a static blueprint of genomic perturbations, they fail to capture how molecular alterations propagate through regulatory networks to reshape dynamic cellular behavior. Consequently, the functional impact of patient-specific genomic alterations on the temporal execution of the cell cycle remains a fundamental challenge in precision oncology.

Systems biology^9^ offers a principled framework to address this gap by integrating mechanistic models with high-dimensional molecular data^10^. In particular, stochastic modeling^11^ provides a natural description of cell cycle transitions, which arise from nonlinear interactions, feedback loops, and threshold-dependent regulatory mechanisms. Such models enable the characterization of emergent dynamical properties that cannot be inferred from individual molecular components or static statistical correlations alone.

Mechanistic modeling of cell cycle regulation has a long tradition in systems biology, establishing the switch-like and irreversible nature of major transitions^12–17^. In particular, models of the G2/M transition have elucidated how CDK-cyclin complexes, phosphatases, and kinases interact through positive feedback loops to ensure robust mitotic entry. However, these foundational studies were largely developed in idealized biochemical contexts or model organisms. More generally, while quantitative models have demonstrated that the system-level impact of perturbations depends on network context rather than individual changes^18^, their application to patient-specific clinical data remains limited.

More recently, computational approaches have explored the integration of molecular alterations into mechanistic and network-based models to study phenotypic heterogeneity in cancer. These efforts range from whole-cell and pathway-scale models linking genotype to phenotype^19^ to systems-level frameworks in which genetic or pharmacological alterations are modeled as network perturbations^20–22^. Nevertheless, most existing approaches rely on data obtained under controlled experimental conditions or focus on signaling pathways, rarely addressing how patient-specific genomic alterations reshape the intrinsic dynamics of core cell cycle regulation.

To bridge this gap, we extend current systems-level approaches by integrating patient-specific copy number alterations into a stochastic, mechanistic model of the human G2/M transition. By instantiating a feedback-driven regulatory framework with human G2/M regulators and quantitatively mapping CNAs as system perturbations, we move beyond descriptive genomics to generate individualized dynamical phenotypes. This approach enables the emergence of patient-specific transition timing and likelihood of timely mitotic commitment, which we then assess for their association with clinical outcome. Breast cancer was selected as a reference case study due to the high prevalence of copy number alterations affecting G2/M cell cycle regulators and the availability of large, well-annotated patient cohorts, enabling robust evaluation of patient-specific dynamical modeling^7,23^.

## Results

### A human-specific stochastic model of the G2/M transition

**Fig. 1:**
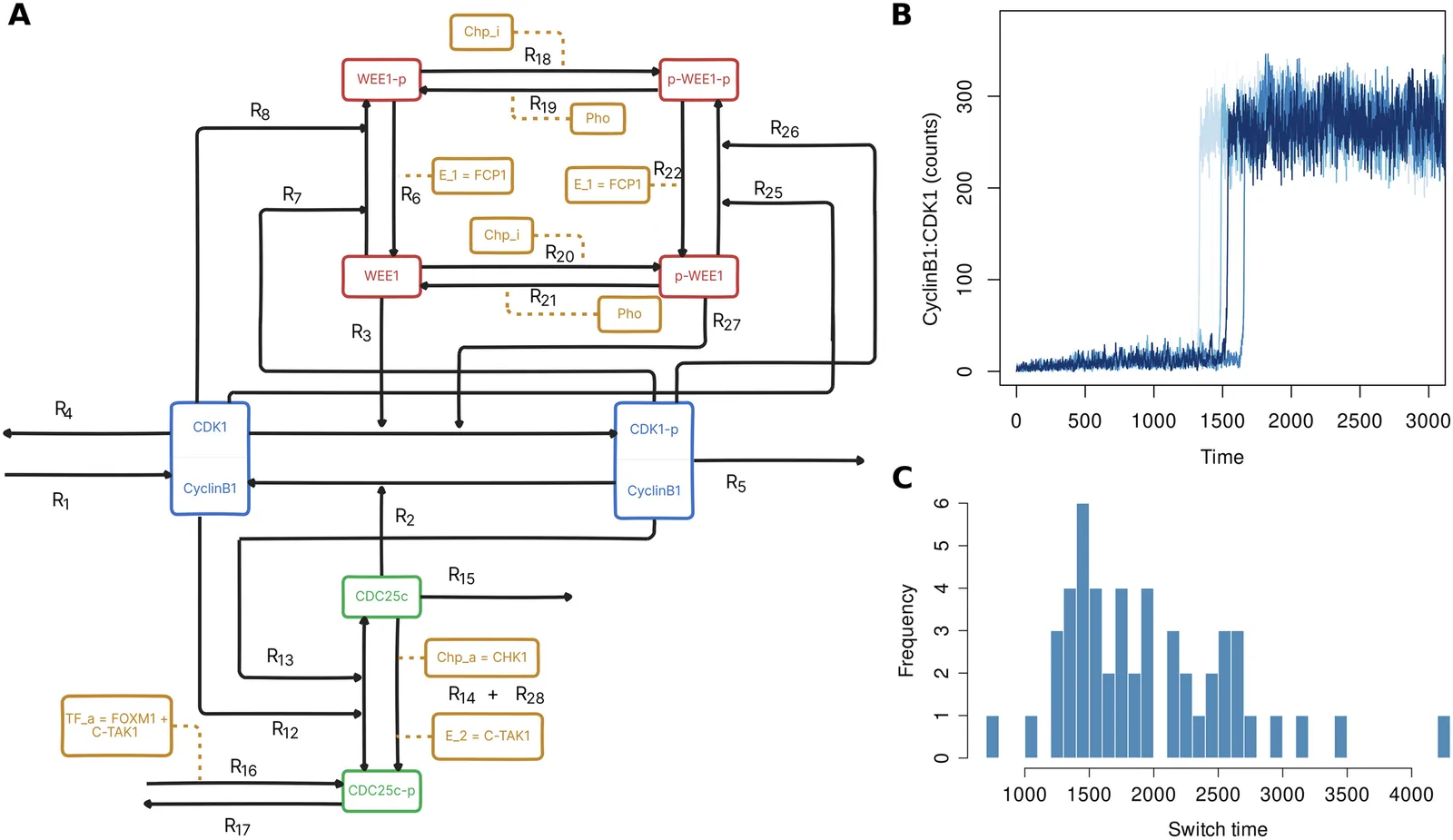
Human-specific stochastic model of the G2/M cell cycle transition. **A** Schematic representation of the human-specific G2/M cell cycle regulatory model. The transition regulator is the CDK1–Cyclin B1 complex, whose activation is controlled by the opposing activities of the activator CDC25C and the inhibitor WEE1. Checkpoint signaling mediated by CHK1 inhibits CDC25C activity. Positive feedback loops couple activation of the transition regulator to both activator activation and inhibitor inhibition, generating a switch-like regulatory architecture. Constitutive enzymatic activities (E1, E2, Pho) represent background kinase and phosphatase processes maintaining steady-state signaling. **B** Representative stochastic trajectory of active CDK1–Cyclin B1 levels over time in the diploid reference configuration (no copy number alterations). The transition regulator exhibits threshold-dependent activation, marking commitment to mitosis under intrinsic stochastic fluctuations. **C** Distribution of switch times across independent stochastic simulations under baseline (diploid) conditions. Switch time is defined as the first time point at which active CDK1–Cyclin B1 exceeds the predefined commitment threshold.

We first developed a stochastic, human-specific model of the G2/M cell cycle transition to provide a mechanistic framework for integrating patient-specific genomic perturbations (Supplementary Information). The model builds upon established feedback-driven representations of cell cycle transitions, in which G2/M commitment emerges from interacting positive feedback loops and checkpoint regulation, as previously described^24^, and instantiates this abstract architecture using regulators of the human G2/M transition (Fig. 1A).

Consistent with its feedback-driven structure, baseline simulations reproduced the expected G2/M transition dynamics, with stochastic variability in transition timing across individual trajectories (Fig. 1B). The distribution of switch times was consistent with a well-defined baseline dynamical regime for mitotic commitment (Fig. 1C).

Sensitivity analysis was performed by varying one parameter or species at a time to assess the influence of individual model components on transition dynamics (Supplementary Fig. 1). This analysis identifies key regulators of transition timing and commitment percentage, showing that perturbations of specific components can induce substantial changes in system behavior. This behavior reflects the threshold-dependent nature of the system, in which cumulative perturbations can induce qualitative changes in transition dynamics, such as switching between successful and failed transitions or between fast and delayed transitions.

### Copy number alterations induce systematic perturbations of G2/M dynamics

**Fig. 2:**
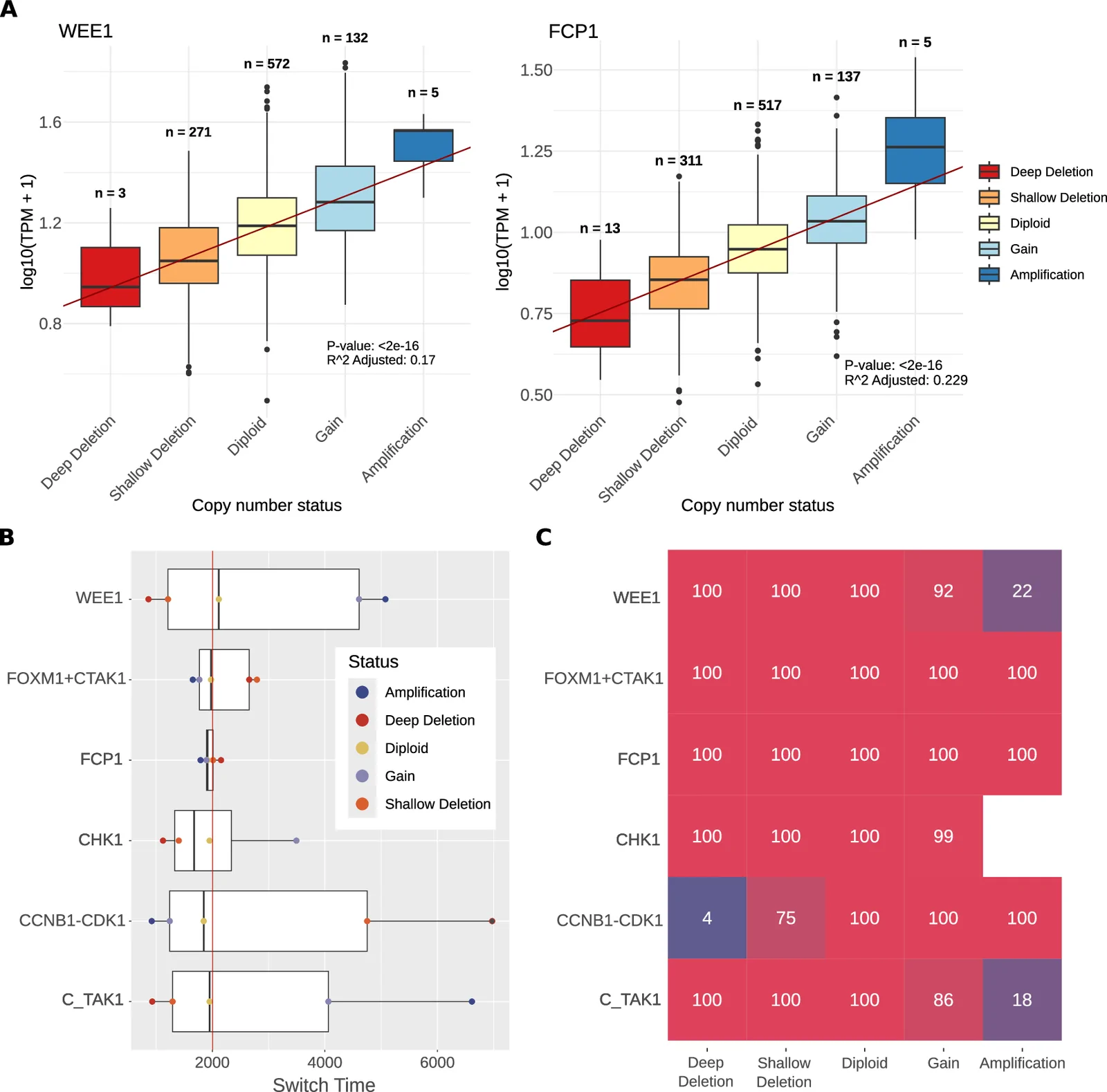
Copy number alterations reshape G2/M transition dynamics. **A** Association between gene-level copy number status and normalized gene expression for representative regulators of the human G2/M transition. Boxplots show log10(TPM + 1) expression levels stratified by copy number category (deep deletion, shallow deletion, diploid, gain, amplification) for selected genes (e.g., WEE1 and FCP1). Red lines indicate linear regression fits, illustrating dosage-dependent expression changes used to parameterize model species. **B** Effect of copy number alterations on model-derived switch time across genes included in the G2/M regulatory module. Horizontal boxplots display the distribution of switch times for each gene under different copy number states. The vertical red line indicates the diploid reference configuration. **C** Switch commitment percentage (fraction of stochastic simulations completing the G2/M transition within the predefined maximum simulation time) across copy number categories for each modeled gene. Values represent the percentage of simulations reaching the commitment threshold within the finite simulation horizon.

We next investigated how somatic copy number alterations affecting G2/M regulators perturb model dynamics. Because the abstract regulatory framework is instantiated using human G2/M regulators, gene-level copy number alterations can be interpreted as quantitative perturbations of model species. Using breast cancer genomic and transcriptomic data, we quantified the relationship between gene copy number status and normalized gene expression for each modeled gene, identifying statistically significant linear associations that enabled quantitative mapping of CNAs to model components (Fig. 2A and Supplementary Fig. 2).

Incorporating CNA-driven perturbations into the human-specific model revealed systematic effects on G2/M transition dynamics. Copy number gains affecting activators or components of the transition regulator resulted in reduced switch times and increased switching commitment percentage, whereas gains affecting inhibitory components, or losses affecting activators, produced delayed transitions and reduced commitment percentage, in some cases preventing transition altogether (Fig. 2B).

As expected for a feedback-driven regulatory system, the dynamical impact of copy number alterations depended on their position within the network rather than on copy number status alone, underscoring the role of mechanistic context in shaping cell cycle dynamics.

### Patient-specific modeling reveals distinct dynamical regimes

Patient-specific simulations were generated for 975 breast cancer patients with available copy number and clinical data by parameterizing the human-specific G2/M model according to individual CNA profiles. For each patient, one hundred stochastic simulations were performed to compute mean switch time and switch commitment percentage, capturing patient-level variability in G2/M transition dynamics.

**Fig. 3:**
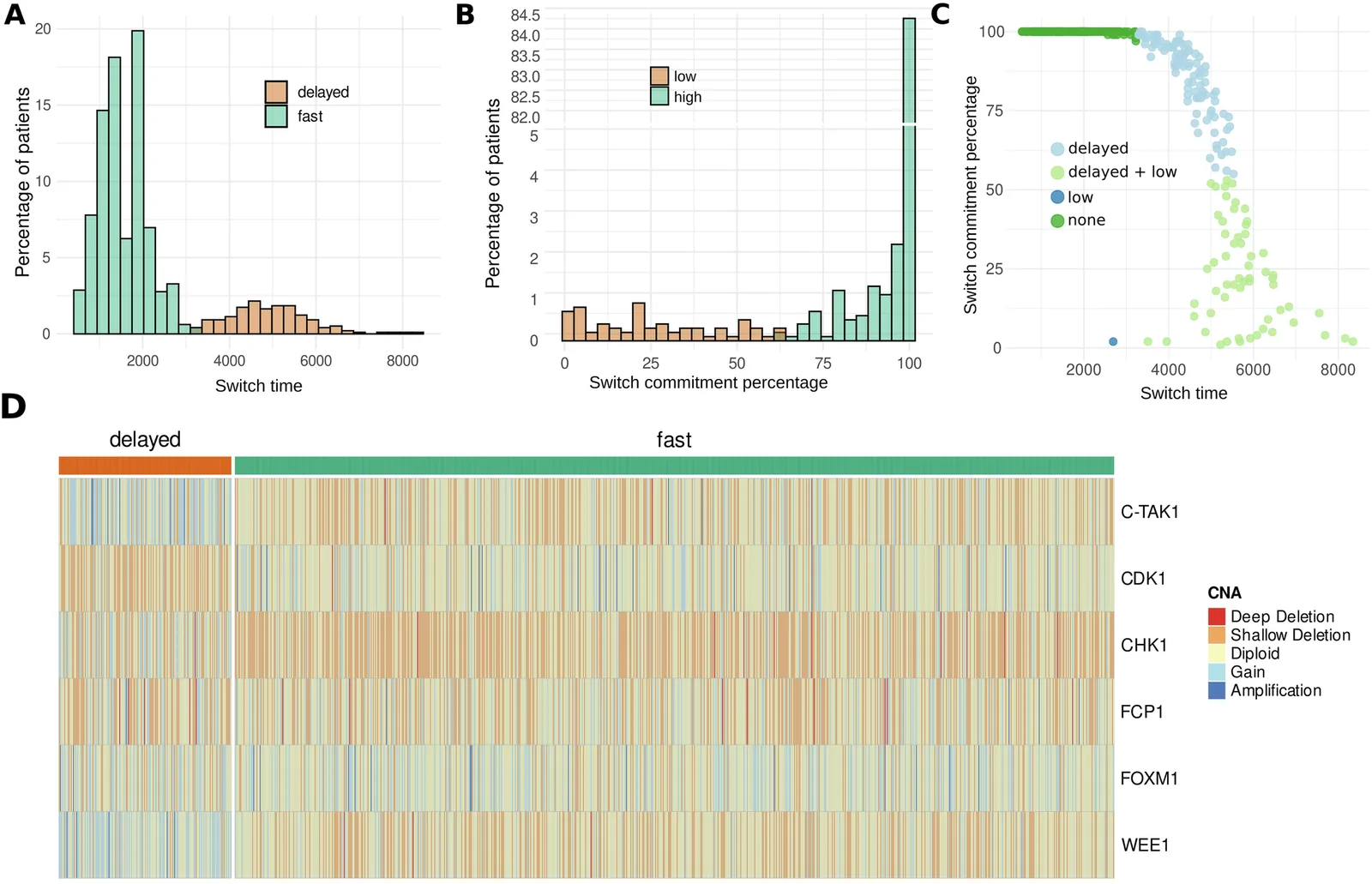
Patient-specific modeling identifi es distinct G2/M dynamical regimes. **A** Distribution of patient-specific mean switch times derived from stochastic simulations of the human-specific G2/M model (*n* = 975). **B** Distribution of switch commitment percentage, defined as the fraction of stochastic simulations completing the G2/M transition within the predefined maximum simulation time. **C** Joint distribution of mean switch time and switch commitment percentage for individual patients. Each point represents a patient-specific model parameterization. Distinct clusters correspond to delayed, delayed with low commitment percentage, low commitment percentage, and baseline-like configurations. **D** Oncoplot representation of gene-level copy number alterations for regulators included in the G2/M module, stratified according to the identified dynamical regimes (delayed versus fast). Copy number categories (deep deletion, shallow deletion, diploid, gain, amplification) are shown for each patient.

The distribution of patient-specific mean switch times exhibited a clear bimodal structure (Fig. 3A), indicating the presence of two distinct dynamical regimes characterized by fast versus delayed G2/M transitions. A comparable bimodal pattern was observed for switch commitment percentage (Fig. 3B), reflecting differences in the efficiency of G2/M commitment under stochastic dynamics across patients. Distributions of mean switch time and switch commitment percentage across molecular subtypes are reported in Supplementary Fig. 3 and do not show evident subtype-specific differences. Consistently, both clusters include patients from all major molecular subtypes (Supplementary Fig. 4).

Clustering patients based on model-derived dynamical metrics separated the cohort into well-defined groups (Fig. 3C). These groups did not correspond to individual copy number events or single-gene alterations (Fig. 3D), but instead emerged from the combined effect of multiple perturbations acting within the regulatory network. In this context, patient stratification reflects differences in the integrated dynamical behavior of the cell cycle control system rather than isolated genomic features.

Together, these results show that patient-specific integration of copy number alterations into a mechanistic G2/M model yields emergent dynamical phenotypes that capture inter-patient heterogeneity and provide a principled basis for downstream clinical association analyses.

### Model-derived dynamical phenotypes stratify patient survival

**Fig. 4:**
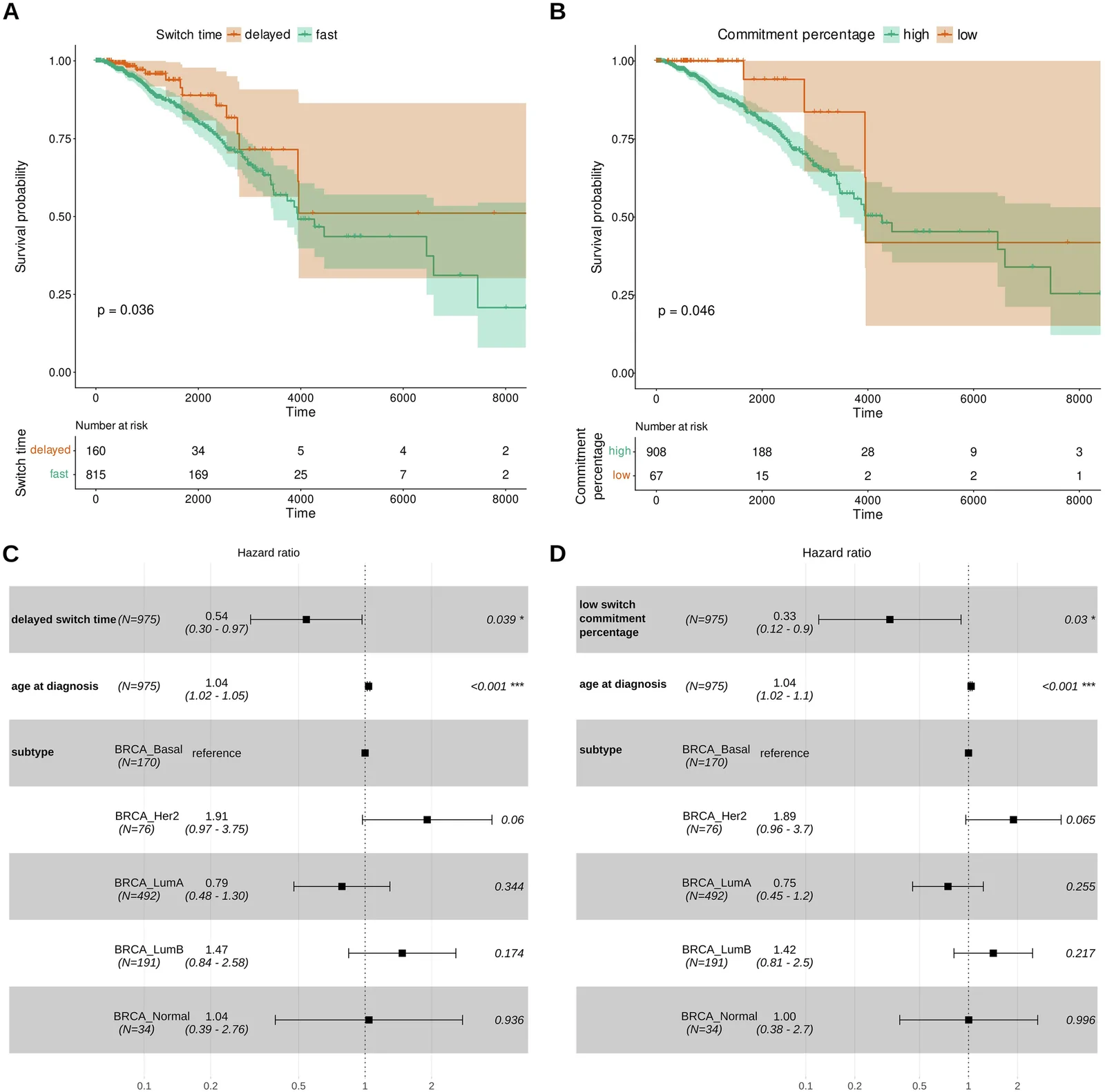
Model-derived G2/M dynamical regimes are associated with overall survival. **A** Kaplan–Meier survival curve stratified by mean switch time-derived dynamical regimes. Patients classified as having delayed G2/M commitment exhibit improved overall survival compared to those with relatively faster commitment dynamics (log-rank *p* = 0.036). Numbers at risk are shown below the plot. **B** Kaplan–Meier survival curves stratified by switch commitment percentage. Patients with a lower likelihood of timely G2/M commitment exhibit improved overall survival compared to those with a higher commitment percentage (log-rank *p* = 0.046). **C** Multivariate Cox proportional hazards model evaluating the association between delayed switch time regime and overall survival, adjusting for age at diagnosis and molecular subtype. Hazard ratios (HR) with 95% confidence intervals are shown. The delayed switch time regime remains significantly associated with survival (HR < 1), independent of covariates. **D** Multivariate Cox proportional hazards model evaluating the association between low switch commitment percentage and overall survival, adjusting for age at diagnosis and molecular subtype. Low commitment percentage remains significantly associated with improved survival (HR < 1), independent of covariates.

We next evaluated whether the dynamical regimes emerging from patient-specific modeling were associated with clinical outcomes. Kaplan–Meier survival analysis revealed significant differences in overall survival between patients belonging to distinct dynamical regimes. In particular, patients characterized by delayed G2/M transitions exhibited significantly improved survival compared to those with faster transitions (Fig. 4A). A consistent separation was observed when survival was analyzed using commitment percentage as the stratifying metric, with lower percentage of commitment to G2/M transition associated with improved survival (Fig. 4B). These findings show that model-derived differences in transition timing and commitment percentage are significantly associated with overall survival across patients. When stratified by molecular subtype, similar survival patterns were observed across subtypes, although statistical power was reduced within individual groups (Supplementary Fig. 5).

To formally assess whether these associations are independent of clinical variables, multivariate Cox proportional hazards models were fitted, including age at diagnosis and molecular subtype as covariates. Model-derived stratification remained significantly associated with overall survival after adjustment (Fig. 4C, D), indicating that the identified dynamical regimes capture prognostically relevant information beyond conventional clinical annotations.

Finally, to evaluate the robustness of these associations to the choice of clustering thresholds, we repeated the Cox analysis using switch time and commitment percentage as continuous variables. In both cases, the association with overall survival remained statistically significant (commitment percentage: HR = 1.015, 95% CI: 1.001–1.029, *p* = 0.031; switch time: HR = 0.9999 per unit increase, 95% CI: 0.9997–1.000, *p* = 0.045), indicating that the results are not sensitive to the specific cutoff choices.

To further contextualize model-derived dynamical features with respect to established proliferation-associated markers, we performed additional analyses incorporating MKI67 expression. The association between dynamical features and overall survival remained statistically significant after adjustment for MKI67, age, and molecular subtype (Supplementary Fig. 6), indicating that these features capture aspects of tumor behavior not fully explained by standard proliferation markers. In addition, we performed hierarchical clustering of patients based on the expression of the 21 OncotypeDx genes (Supplementary Fig. 7). This analysis did not reveal a clear segregation of samples according to the model-derived clusters, suggesting that the dynamical stratification is not simply a reflection of transcriptomic clustering based on proliferation-associated gene sets.

Together, these findings demonstrate that patient-specific dynamical features emerging from a mechanistic G2/M cell cycle model are associated with differences in overall survival, supporting the use of systems-level modeling to link genomic perturbations with clinically meaningful tumor phenotypes.

## Discussion

In this study, we developed a stochastic, human-specific model of the G2/M cell cycle transition and used it to investigate how patient-specific copy number alterations perturb cell cycle dynamics. By instantiating a feedback-driven regulatory framework with human G2/M regulators and integrating gene-level copy number data, we translated static genomic alterations into dynamic phenotypes, capturing transition timing and likelihood of timely mitotic commitment. Our results show that these model-derived dynamical features capture inter-patient heterogeneity and are associated with differences in overall survival in breast cancer.

A central observation of this work is that the dynamical impact of copy number alterations depends on their position within the regulatory network rather than on copy number status alone. This behavior is consistent with general principles of feedback-driven regulatory systems, in which nonlinear interactions and positive feedback loops shape system-level responses to perturbations rather than individual molecular changes in isolation. Such architectures are well known to give rise to switch-like behavior, bistability, and context-dependent sensitivity to perturbations in cell cycle and signaling networks^12–17^. In this framework, identical copy number gains or losses can produce distinct dynamical outcomes depending on whether they affect activators, inhibitors, or components of the transition regulator, highlighting the importance of mechanistic context when interpreting cancer genomic alterations.

At the patient level, integrating multiple copy number alterations within a unified dynamical framework revealed the existence of distinct G2/M dynamical regimes. These regimes were not directly inferred from individual genomic events but emerged from the combined effect of multiple perturbations acting within the nonlinear regulatory network. Patients characterized by delayed or less efficient G2/M transitions exhibited improved overall survival. Within the modeling framework, reduced switch commitment percentage reflects a lower likelihood of completing the G2/M transition within the simulated time horizon under stochastic perturbations, a behavior consistent with reduced proliferative efficiency. While this interpretation remains model-based, it provides a plausible mechanistic explanation for the observed survival association. In this context, survival analysis serves as a functional validation of the model-derived phenotypes rather than as a primary clinical objective. Notably, the association between slower cell cycle progression and improved prognosis has been independently reported in breast cancer using orthogonal approaches, such as measurements of cell cycle traverse rate^25^.

More broadly, this work illustrates the value of systems-level modeling for bridging cancer genomics and functional cellular behavior. Rather than treating genomic alterations as independent predictors, our approach uses them as quantitative perturbations of a dynamic system, allowing higher-level phenotypes to emerge from network interactions. This process-centric perspective complements gene-centric statistical analyses and aligns with previous efforts demonstrating how mechanistic models can connect genotype to phenotype through system dynamics^19,26^.

Several limitations of the present study should be acknowledged. First, the model focuses exclusively on the G2/M transition and does not account for other cell cycle phases or their coupling to additional cellular processes such as DNA repair, apoptosis, senescence, or metabolism. Consequently, the inferred dynamical phenotypes represent only one component of overall tumor proliferation dynamics. Second, patient-specific perturbations are derived solely from somatic copy number alterations, while other classes of genomic variation, such as point mutations and small indels, remain unaddressed. While this may limit the model’s applicability to pathways dominated by sequence-level drivers, the G2/M transition in breast cancer represents an ideal case study for a CNA-centric approach. Indeed, the core regulators modeled here (CDK1, CCNB1, WEE1, CDC25C, FOXM1, C-TAK1, FCP1, and CHK1) exhibit remarkably low mutation frequencies (typically <1%) in the TCGA BRCA cohort, whereas they are frequently subject to dosage shifts via copy number gains and losses (Supplementary Fig. 8). Third, copy number alterations are modeled as static, gene-level perturbations and do not capture intra-tumor heterogeneity, clonal evolution, or temporal changes in genomic architecture. In addition, baseline parameter values are inherited from previously established models and are not estimated from patient-specific kinetic data. Sensitivity analysis shows that relatively small perturbations can lead to substantial changes in transition timing or even prevent the transition, reflecting the threshold-dependent behavior of feedback-driven, bistable systems, as previously characterized in the underlying modeling framework^24^. In this context, quantitative outputs such as switch time and commitment percentage should not be interpreted as precise measurements, but rather as indicators of qualitative differences in system behavior. Finally, survival associations were evaluated within a single cohort, and external validation in independent datasets will be required to assess the generalizability of the observed relationships.

Despite these limitations, the proposed framework provides a principled and extensible approach for integrating cancer genomic data into mechanistic models of cell cycle regulation. Future extensions could explore the incorporation of additional layers of genomic variation, including somatic mutations and complex structural alterations, model interactions between multiple cell cycle phases, or simulate therapeutic perturbations targeting G2/M control. More generally, this study demonstrates how stochastic, mechanistic modeling can transform static genomic alterations into dynamic, interpretable phenotypes, supporting the use of systems biology approaches to investigate the functional consequences of tumor heterogeneity.

## Methods

### Human-specific G2/M cell cycle model

We developed a stochastic, mechanistic model of the human G2/M cell cycle transition based on the feedback-driven regulatory framework we previously proposed in ref. ^24^, in which commitment to a transition emerges from nonlinear interactions between activators, inhibitors, and checkpoint regulation. Following the classification of transition regulators for specific cell cycle phases and organisms, we instantiated this abstract architecture for the human G2/M transition.

Specifically, the CDK1-Cyclin B1 complex was selected as the transition regulator, CDC25C as the activator^26–28^, and WEE1 as the inhibitor^27–29^, with checkpoint control on CDC25C mediated by CHK1 (Fig. 1A). Additional regulatory components included in the model were selected to complete a minimal and biologically consistent representation of the human G2/M transition, based on established experimental evidence in the literature.

The model explicitly accounts for the dual checkpoint controls exerted on both inhibitors and activators. Specifically, it incorporates CHK1 as checkpoint regulator of the activator side, reflecting the checkpoint’s ability to halt progression^6,30–33^. Central to this transition is the inclusion of positive feedback loops that control the entry into mitosis, where the activation of the transition regulator promotes both activator activation and inhibitor inhibition, forming positive feedback loops that generate robust, threshold-dependent G2/M transition dynamics^34,35^. To capture the protein fluctuations observed during the cell cycle, the model includes the transcription factor FOXM1, which directly regulates the periodic transcription of CDC25C^36^.

Moreover, the basal enzymatic state relies on the continuous background activity of constitutive enzymes to sustain steady-state signaling. As supported by the literature, this background activity is mediated by kinases such as CTAK1^37,38^, and phosphatases, including FCP1^39^ and those represented by the variable Pho. Various studies have demonstrated the presence of both active and inactive forms of WEE1 during the G2/M transition (indicated as WEE1 and WEE1-p, respectively, in Fig. 1A). Although a hyperactive form of WEE1 has not yet been identified in humans (represented by the p-WEE1 and p-WEE1-p forms in Fig. 1A), our model incorporates this functional state to preserve the feedback structure of the regulatory module. This approach aligns with the architecture described in other eukaryotic systems^40^.

Baseline kinetic laws and parameter values were inherited from the feedback-driven transition model in ref. ^24^ and adapted to the human G2/M regulatory architecture. These parameters define a reference dynamical regime characterized by a well-defined switch-like transition and are not interpreted as measured kinetic constants. Rather, they serve as a baseline from which relative perturbations induced by genomic alterations can be evaluated. Full reaction schemes, kinetic laws, and baseline parameter values are reported in the Supplementary Information.

All molecular species are represented as discrete molecule counts rather than concentrations, enabling explicit stochastic simulation of intrinsic noise effects. Among the transcriptional control configurations described in ref. ^24^, we focused on the STOP-like configuration, in which transcriptional regulation acts on the activator. This configuration reflects the stringent checkpoint control characteristic of the G2/M transition and provides a suitable framework for integrating patient-specific genomic perturbations.

### Stochastic simulations and model outputs

The model was implemented in the BlenX language^41^ and simulated within the Beta Workbench environment^42^, which provides an efficient variant of the Gillespie exact stochastic simulation algorithm for continuous-time Markov processes^43^. This setup enables direct stochastic simulation of feedback-driven cell cycle transition dynamics. The BlenX model code used in this study is provided in the Supplementary Information.

For each model configuration, 100 independent stochastic simulations were performed. Two quantitative outputs were extracted: (i) switch time, defined as the first time point at which the active transition regulator exceeded a predefined threshold (150) indicating irreversible commitment to mitosis and computed by averaging across simulations; (ii) switch commitment percentage, defined as the fraction of stochastic simulations in which the transition threshold was reached within the predefined maximum simulation time (10,000), thereby quantifying the probability of timely mitotic commitment under stochastic dynamics.

### Breast cancer data and patient cohort

Somatic copy number alteration data were obtained from the cBioPortal for Cancer Genomics^44^, while gene expression data were retrieved from the recount3 database^45^. Both datasets correspond to samples from the TCGA Breast Invasive Carcinoma (PanCancer Atlas) cohort^46^. Gene-level CNA profiles and normalized RNA-seq expression values were used for downstream analyses. Overall survival for BRCA patients was defined as the time from diagnosis to death or last follow-up using TCGA clinical data available from ref. ^47^.

Only primary tumor samples were retained, resulting in 983 breast cancer patients with CNA data, including 975 with both CNA and clinical data. Cohort characteristics are summarized in the Supplementary Data 1, 2, and 3.

### Mapping copy number alterations to model perturbations

Patient-specific perturbations were derived from somatic copy number alterations. Other classes of genomic variation, including point mutations, small indels and structural variants, were not considered.

The analysis was restricted to genes included in the mechanistic G2/M model. For each gene, the association between discrete copy number status and normalized log-transformed gene expression was assessed using linear regression across patients. Genes exhibiting a statistically significant association (*P* < 0.01) were used to parameterize model perturbations, with regression coefficients employed to scale the corresponding model species and parameters. For all genes retained in the analysis, the association between copy number and expression was highly significant (*P* < 2 × 10^−^^16^), indicating a strong gene dosage-expression relationship. Given the limited number of tested genes and the strength of these associations, multiple testing correction would not affect the set of genes used for model parameterization.

To further assess the relationship between mRNA expression and protein abundance, we analyzed matched proteomic data for a subset of TCGA samples. Mass spectrometry-based measurements were used as the primary source of protein abundance data and were available for the majority of the modeled genes, while RPPA data were used to increase coverage where mass spectrometry measurements were not available. Across the analyzed genes, we observed generally consistent positive relationships between mRNA and protein levels (Supplementary Fig. 9), supporting the use of transcriptomic data to represent relative gene dosage-driven perturbations in the model.

The CNA–expression relationship was estimated without adjustment for molecular subtype, as the resulting coefficients were used to parameterize patient-specific models rather than to estimate subtype-independent effects. To assess the impact of this choice, we repeated the regression including molecular subtype as a covariate. This yielded highly similar coefficients (mean absolute difference ≈ 0.01), while the associations remained highly statistically significant.

A diploid copy number state was used as the baseline condition, corresponding to a reference species quantity of 500 molecules^24^. This diploid configuration serves as a dynamical reference state for expressing relative shifts induced by patient-specific CNAs and is not intended to represent a typical tumor condition. Deviations from diploidy were translated into proportional changes in species abundance based on the fitted regression models. In the model, most species are associated with a single gene. A single exception is the transcriptional regulator component (TFₐ), which is influenced by multiple genes (e.g., FOXM1 and C-TAK1). For this component, a conservative mapping strategy was adopted by selecting the lowest scaled value among the contributing genes to represent a limiting regulatory effect. This approach avoids overestimating the impact of copy number alterations while maintaining a simple and interpretable mapping between genomic perturbations and model components.

### Patient-specific simulations

For each patient, model species quantities were adjusted according to the individual CNA profile, generating a patient-specific instance of the human G2/M model. Each patient-specific model was simulated 100 times, and the mean switch time and switch commitment percentage were calculated to characterize individualized G2/M transition dynamics.

The complete set of patient-specific model parameterizations derived from individual CNA profiles is reported in Supplementary Data 4.

### Patient stratification and survival analysis

Patients were stratified into two groups based on model-derived dynamical metrics using k-means clustering (*k* = 2), applied separately to mean switch time and switch commitment percentage. The resulting groups correspond to distinct G2/M dynamical regimes identified by patient-specific modeling.

Differences in survival between patient groups were assessed using Kaplan-Meier analysis, with statistical significance (*P* < 0.05) evaluated by the log-rank test. Multivariate Cox proportional hazards models were used to examine associations with survival while adjusting for age at diagnosis and molecular subtype.

To contextualize model-derived features with respect to proliferation-associated markers, additional analyses were performed, including MKI67 expression^48^ as a covariate in Cox proportional hazards models. Furthermore, hierarchical clustering based on the expression of the 21 OncotypeDx^49^ genes was performed as a descriptive comparison with model-derived stratification.

Analyses were performed in R using the *survival* and *survminer* packages^50^.

## Supplementary information

Supplementary information

Supplementary Data 1

Supplementary Data 2

Supplementary Data 3

Supplementary Data 4

## Data Availability

The datasets generated and analyzed during the current study are publicly available at https://github.com/cibiobcg/G2-M_CellCycleModel_CNA. These data constitute the minimal dataset required to interpret, replicate, and build upon the findings reported in this study.

## Code availability

All model code and analysis scripts used in this study are publicly available at https://github.com/cibiobcg/G2-M_CellCycleModel_CNA.
