# Supplementary material for: Patient-specific modeling of G2/M cell cycle dynamics reveals prognostic dynamical regimes driven by copy number alterations
Source: NPJ Syst Biol Appl. 2026 Jun 8;12:138. doi: 10.1038/s41540-026-00766-4 (PMC13578713; doi:10.1038/s41540-026-00766-4)
Supplement: Supplementary file 1 [file 41540_2026_766_MOESM1_ESM.pdf]

Patient-specific modeling of G2/M cell cycle dynamics reveals prognostic dynamical regimes driven by copy number alterations

Supplementary Information

Andrea Tonina[1] and Alessandro Romanel[1,*]

[1]Department of Cellular, Computational and Integrative Biology (CIBIO), University of Trento, Trento, Italy
[*]Correspondence: alessandro.romanel@unitn.it

Contents

1 Model Equations and Parameters 2

2 Blenx Model 3

3 Mass balances for Species 5

4 Sensitivity Analysis 6

5 CNA-expression regressions 7

6 Distributions of switch time and commitment percentage across BRCA subtypes 8

7 Distributions of switch time and commitment percentage across BRCA subtypes and clusters 9

8 Kaplan-Meier survival analysis across BRCA subtypes 10

9 Cox proportional hazards analysis adjusting for MKI67 expression 11

10 Heatmaps of 21 OncotypeDx genes 12

11 TCGA BRCA oncoplots for CNAs and point mutations 13

12 Comparison between Protein and mRNA expression 14

13 Supplementary Data 1-4 15

1 Model Equations and Parameters

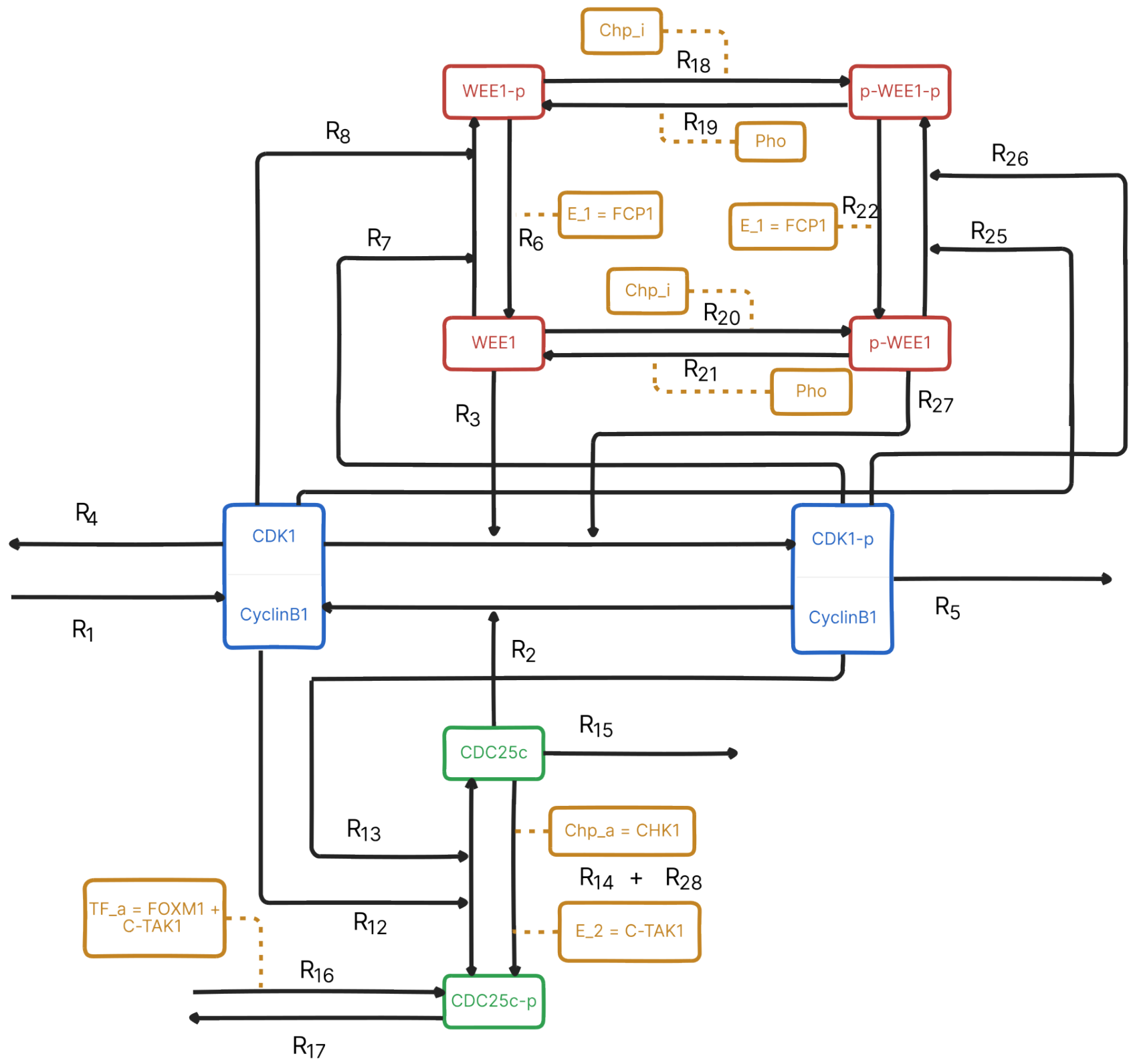

$R_1 = \frac{kms}{\alpha}$	$R_2 = \frac{kma \cdot \alpha \cdot CDC25C \cdot CyclinB1:CDK1p}{jma+(\alpha \cdot CyclinB1:CDK1p)}$	$R_3 = \frac{kmi \cdot \alpha \cdot WEE1 \cdot CyclinB1:CDK1}{jmi+(\alpha \cdot CyclinB1:CDK1)}$
$R_4 = kmd \cdot CyclinB1 : CDK1$	$R_5 = kmd_1 \cdot CyclinB1 : CDK1p$	$R_6 = \frac{kma \cdot \alpha \cdot FCP1 \cdot WEE1p)}{jwa+(\alpha \cdot WEE1p}$
$R_7 = \frac{kwi \cdot \alpha \cdot perc \cdot CyclinB1:CDK1p \cdot WEE1}{jwi+(\alpha \cdot WEE1)}$	$R_8 = \frac{kwi \cdot \alpha \cdot CyclinB1:CDK1 \cdot WEE1}{jwi+(\alpha \cdot WEE1)}$	$R_{12} = \frac{kca \cdot \alpha \cdot CyclinB1:CDK1CDC25Cp}{jca+(\alpha \cdot CDC25Cp)}$
$R_{13} = \frac{kca \cdot \alpha \cdot perc \cdot CyclinB1:CDK1p \cdot CDC25Cp}{jca+(\alpha \cdot CDC25Cp)}$	$R_{14} = \frac{kci \cdot \alpha \cdot C-TAK1 \cdot CDC25C}{jci+(\alpha \cdot CDC25C)}$	$R_{15} = kcd \cdot CDC25C$
$R_{16} = kcs \cdot FOXM1 : C - TAK1$	$R_{17} = kcd_1 \cdot CDC25Cp$	$R_{18} = \frac{kcp_7 \cdot \alpha \cdot Chp_i \cdot WEE1p)}{jcp_7+(\alpha \cdot WEE1p)}$
$R_{19} = \frac{kcp_6 \cdot \alpha \cdot Pho \cdot pWEE1p}{jcp_6+(\alpha \cdot pWEE1p)}$	$R_{20} = \frac{kcp_1 \cdot \alpha \cdot Chp_i \cdot WEE1}{jcp_1+(\alpha \cdot WEE1)}$	$R_{21} = \frac{kcp_2 \cdot \alpha \cdot Pho \cdot pWEE1}{jcp_2+(\alpha \cdot pWEE1)}$
$R_{22} = \frac{kcp_5 \cdot \alpha \cdot FCP1 \cdot pWEE1p}{jcp_5+(\alpha \cdot pWEE1p)}$	$R_{25} = \frac{kcp_3 \cdot \alpha \cdot CyclinB1:CDK1 \cdot pWEE1}{jcp_3+(\alpha \cdot pWEE1)}$	$R_{26} = \frac{kcp_4 \cdot \alpha \cdot perc \cdot CyclinB1:CDK1p \cdot pWEE1}{jcp_4+(\alpha \cdot pWEE1)}$
$R_{27} = \frac{kmi_1 \cdot \alpha \cdot pWEE1 \cdot CyclinB1:CDK1}{jmi+(\alpha \cdot CyclinB1:CDK1)}$	$R_{28} = \frac{kcp_8 \cdot \alpha \cdot CHK1 \cdot CDC25C}{jcp_8+(\alpha \cdot CDC25C)}$	

Reaction rates for synthesis and degradation are governed by the law of mass action, whereas all other biochemical transitions follow Michaelis-Menten kinetics. Within this framework, parameters prefixed with k denote catalytic constants (expressed in min^{-1}), while those prefixed with j represent dimensionless Michaelis constants. Furthermore, because the system is modeled using explicit molecule counts rather than concentrations, we employ a scaling factor α to represent the system size (cell volume), following the methodology established by Mura and Csikász-Nagy (2008).

$kms = 0.004$	$kma = 0.5$	$jma = 1.0$	$kmi = 0.5$
$jmi = 1.0$	$kmd = 0.002$	$kmd_1 = 0.002$	$jwa = 0.1$
$kwi = 2.0$	$kca = 2.0$	$jca = 0.1$	$kci = 0.2$
$jci = 0.1$	$kmi_1 = 2.0$	$jwi = 0.1$	$kcs = 0.01$
$kcd = 0.01$	$kcd_1 = 0.01$	$jcp_1 = 2.0$	$jcp_2 = 0.1$
$jcp_3 = 0.1$	$jcp_4 = 0.1$	$jcp_5 = 0.1$	$jcp_6 = 0.1$
$jcp_7 = 0.1$	$jcp_8 = 0.1$	$kcp_1 = 0.1$	$kcp_2 = 0.2$
$kcp_3 = 2.0$	$kcp_4 = 2.0$	$kcp_5 = 0.2$	$kcp_6 = 0.2$
$kcp_7 = 0.2$	$kcp_8 = 0.1$	$perc = 0.01$	$\alpha = \frac{1}{500}$

This table shows the values associated to basal parameters of the previous table.

2 Blenx Model

```
[ steps = 20000 , delta = 0.5 ]

// TR
let cyclinB1_CDK1_p : bproc = #(x,cyclinB1_CDK1_p) [ nil ];
let cyclinB1_CDK1 : bproc = #(x_p,cyclinB1_CDK1) [ nil ];

// addjunction
when (cyclinB1_CDK1 :: r_1) new(1);

//deletions
when (cyclinB1_CDK1_p :: r_5) delete(1);
when (cyclinB1_CDK1 :: r_4) delete(1);

// movements
when (cyclinB1_CDK1 :: r_3) split(cyclinB1_CDK1_p, Nil);
when (cyclinB1_CDK1 :: r_27) split(cyclinB1_CDK1_p, Nil);
when (cyclinB1_CDK1_p :: r_2) split(cyclinB1_CDK1, Nil);

// ACTIVATOR
let CDC25C_p : bproc = #(y, CDC25C_p) [ nil ];
let CDC25C : bproc = #(y_p, CDC25C) [ nil ];

// addjunction
when (CDC25C_p :: r_16) new(1);

// deletions
when (CDC25C :: r_15) delete(1);
when (CDC25C_p :: r_17) delete(1);

// movements
```

```
when (CDC25C_p :: r_13) split(CDC25C, Nil);
when (CDC25C_p :: r_12) split(CDC25C, Nil);
when (CDC25C :: r_14) split(CDC25C_p, Nil);
when (CDC25C :: r_28) split(CDC25C_p, Nil);


// INHIBITORS
let WEE1_p : bproc = #(z, WEE1_p) [ nil ];
let WEE1 : bproc = #(z_p, WEE1) [ nil ];
let p_WEE1_p : bproc = #(p_z, WEE1) [ nil ];
let p_WEE1 : bproc = #(p_z_p, WEE1_p) [ nil ];

// movements
when (WEE1_p :: r_6) split(WEE1, Nil);
when (WEE1 :: r_8) split(WEE1_p, Nil);
when (WEE1 :: r_7) split(WEE1_p, Nil);
when (WEE1_p :: r_18) split(p_WEE1_p, Nil);
when (p_WEE1_p :: r_19) split(WEE1_p, Nil);
when (WEE1 :: r_20) split(p_WEE1, Nil);
when (p_WEE1 :: r_21) split(WEE1, Nil);
when (p_WEE1_p :: r_22) split(p_WEE1, Nil);
when (p_WEE1 :: r_25) split(p_WEE1_p, Nil);
when (p_WEE1 :: r_26) split(p_WEE1_p, Nil);

//others
let FOXM1_plus_CTAK1 : bproc = #(d, FOXM1_plus_CTAK1) [ nil ];
let FCP1 : bproc = #(f, FCP1) [ nil ];
let C_TAK1 : bproc = #(g, C_TAK1) [ nil ];
let CHK1 : bproc = #(h, CHK1) [ nil ];
let Chp_i : bproc = #(j, Chp_i) [ nil ];
let Pho : bproc = #(k, Pho) [ nil ];

run 500 WEE1 || 500 FOXM1_plus_CTAK1 || 500 FCP1 || 500 C_TAK1 || 500 CHK1 || 500 Chp_i || 500 Pho
```

Blenx model, this code is also present in the file .prog in the GitHub folder.

3 Mass balances for Species

These are the Mass balances equations for the species based by utilizing the rates, which already contain in the concentrations of the species:

- $\frac{d(CyclinB1{:}CDK1p)}{dt} = R_3 + R_{27} - R_2 - R_5$
- $\frac{d(CyclinB1{:}CDK1)}{dt} = R_1 + R_2 - R_4 - R_{27} - R_5$
- $\frac{dCDC25C}{dt} = R_{12} + R_{13} - R_{28} - R_{14} - R_{15}$
- $\frac{dCDC25Cp}{dt} = R_{16} + R_{14} + R_{28} - R_{17} - R_{13} - R_{12}$
- $\frac{dWEE1}{dt} = R_6 + R_{21} - R_8 - R_7 - R_{20}$
- $\frac{dWEE1p}{dt} = R_8 + R_7 + R_{19} - R_6 - R_{18}$
- $\frac{dpWEE1}{dt} = R_{20} + R_{22} - R_{21} - R_{26} - R_{25}$
- $\frac{dpWEE1p}{dt} = R_{18} + R_{26} + R_{25} - R_{19} - R_{22}$
- $\frac{d(FOXM1{:}C-TAK1)}{dt} = 500$
- $\frac{dC-TAK1}{dt} = 500$
- $\frac{dCHK1}{dt} = 500$
- $\frac{dFCP1}{dt} = 500$
- $\frac{dChp_i}{dt} = 500$
- $\frac{dPho}{dt} = 500$

4 Sensitivity Analysis

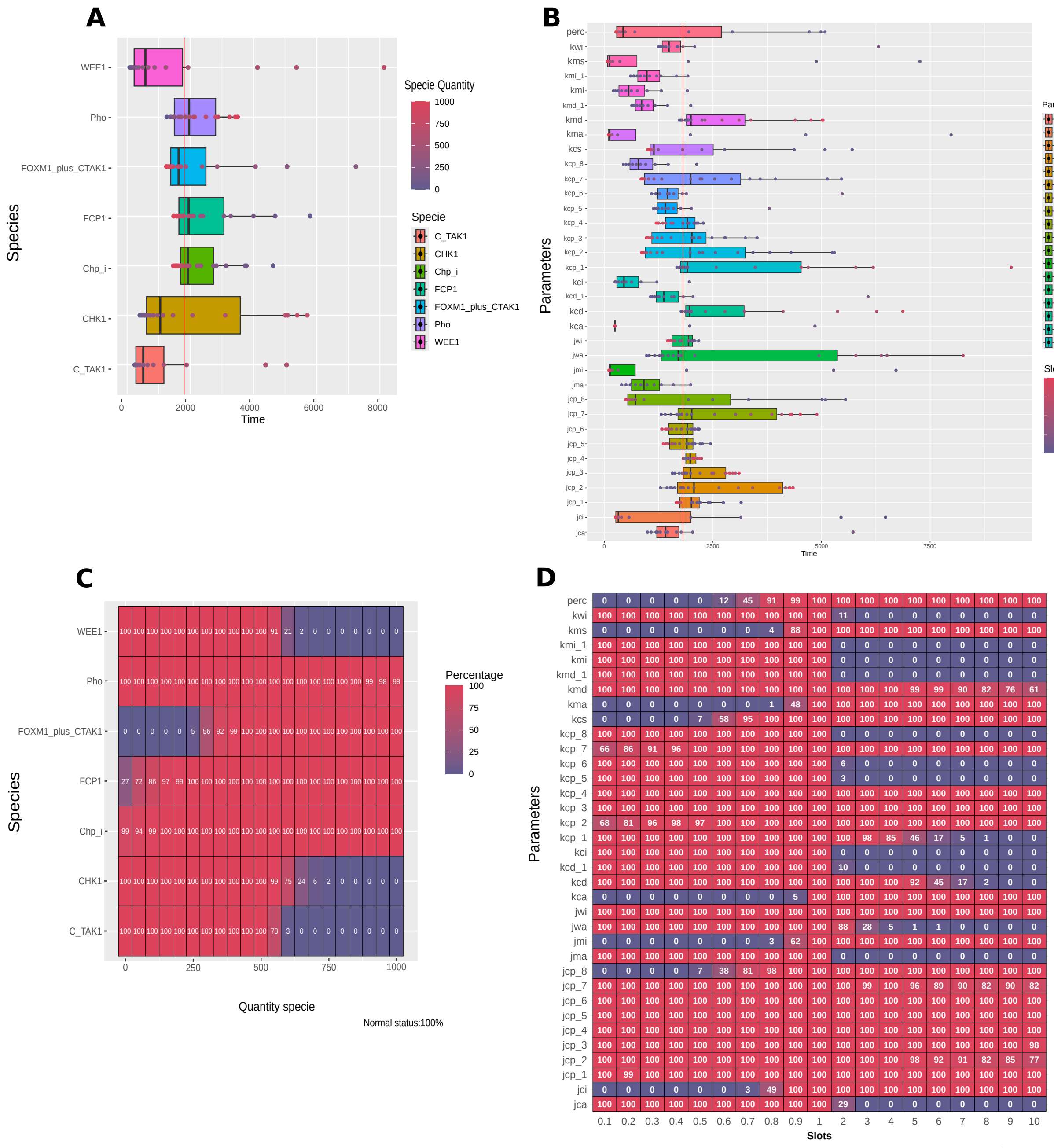

Supplementary Fig. 1. Sensitivity analysis for model species and parameters. To evaluate local sensitivity, species initial values were varied from 0 to 1,000 (in increments of 50), while parameters were scaled from 10% to 100% of their baseline value (10% increments) and from 2- to 10-fold their baseline value (integer increments). A one-at-a-time approach was used, running 100 independent simulations per configuration. The results are organized into four panels: (a, c) species sensitivity and (b, d) parameter sensitivity. In panels (a) and (b), the average switching time (x-axis) is plotted against each variable (y-axis); boxplots illustrate the distribution of switching times, overlaid with individual data points. Panels (c) and (d) utilize heatmaps to display the switching percentage across the tested quantities (x-axis) for each variable (y-axis), scaling from low percentages (violet) to high percentages (red).

5 CNA-expression regressions

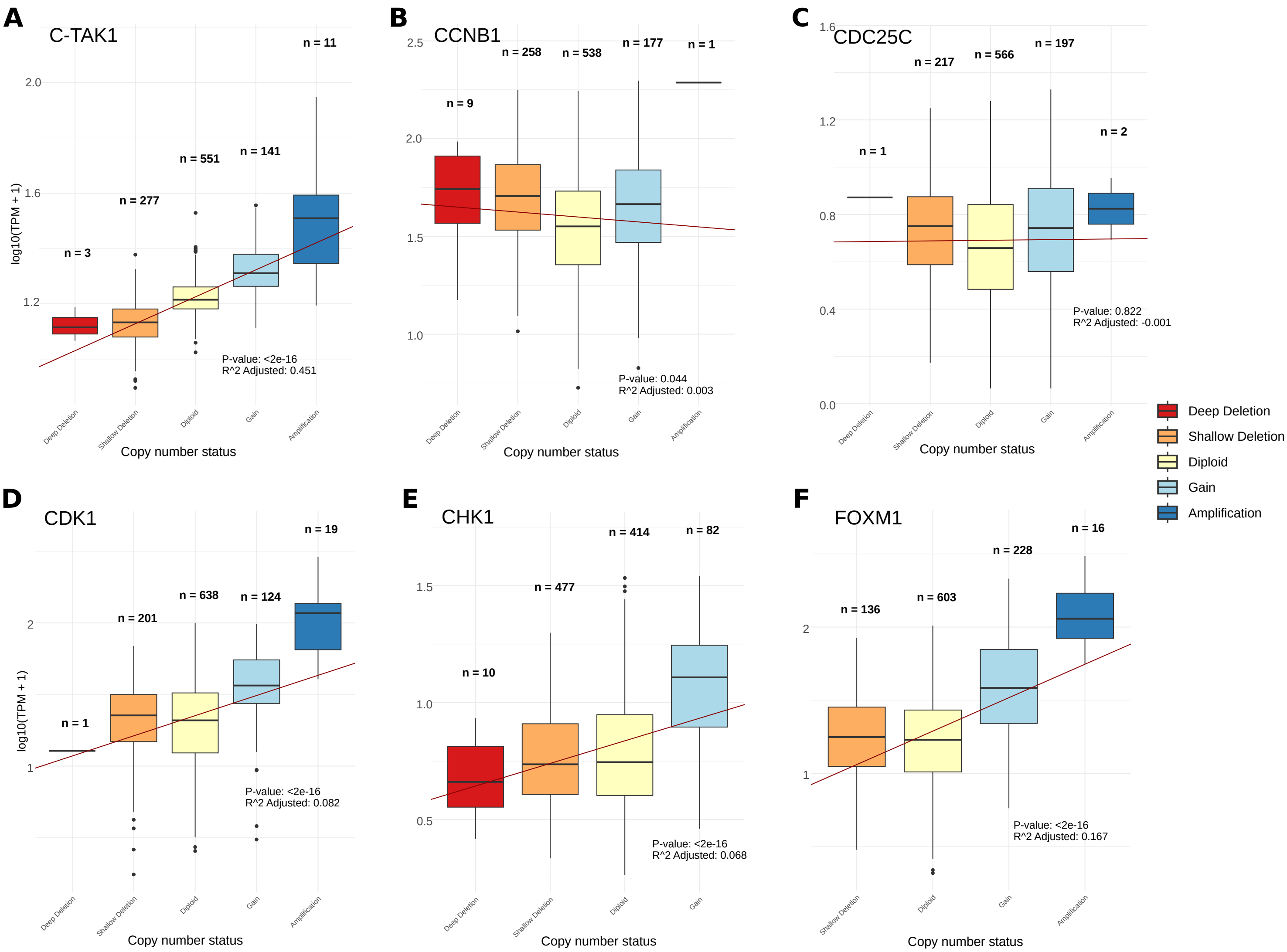

Supplementary Fig. 2. Barplots representing the correlation between CNAs and gene expression levels. Linear regression analysis revealed no observed impact for CDC25C and CCNB1, as evidenced by non-significant p-values. Conversely, the remaining genes exhibited statistically significant linear correlations ($p < 2.2 \cdot 10^{-16}$), with adjusted R^2 values of 0.08 (CDK1), 0.07 (CHK1), 0.23 (FCP1), 0.17 (FOXM1), 0.45 (C-TAK1), and 0.17 (WEE1).

6 Distributions of switch time and commitment percentage across BRCA subtypes

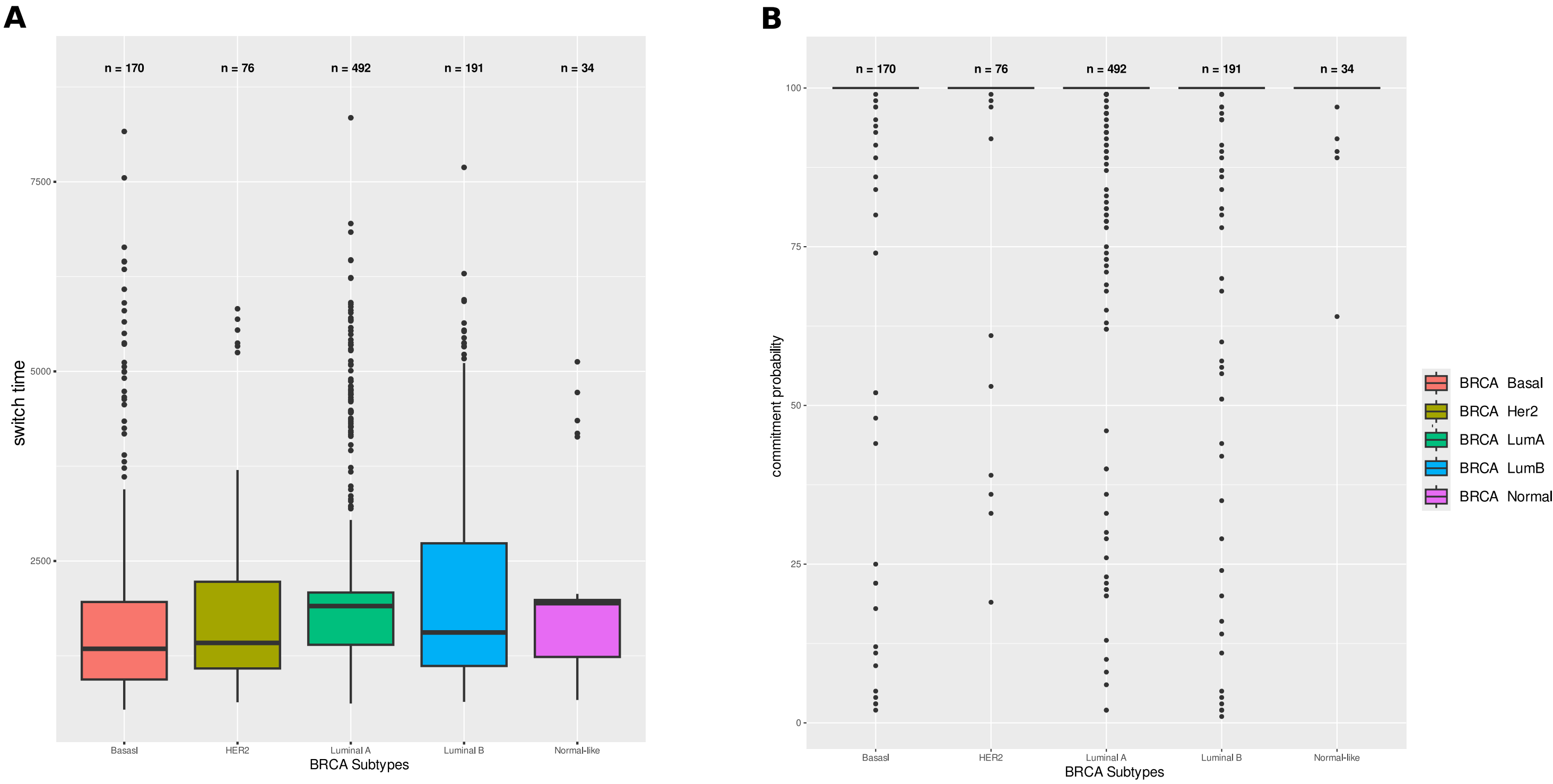

Supplementary Fig. 3. Distributions of patient metrics across subtypes. Figure A displays the mean switch time to demonstrate that all subtypes are adequately represented in the cohort. Figure B presents the same analysis for commitment probability.

7 Distributions of switch time and commitment percentage across BRCA subtypes and clusters

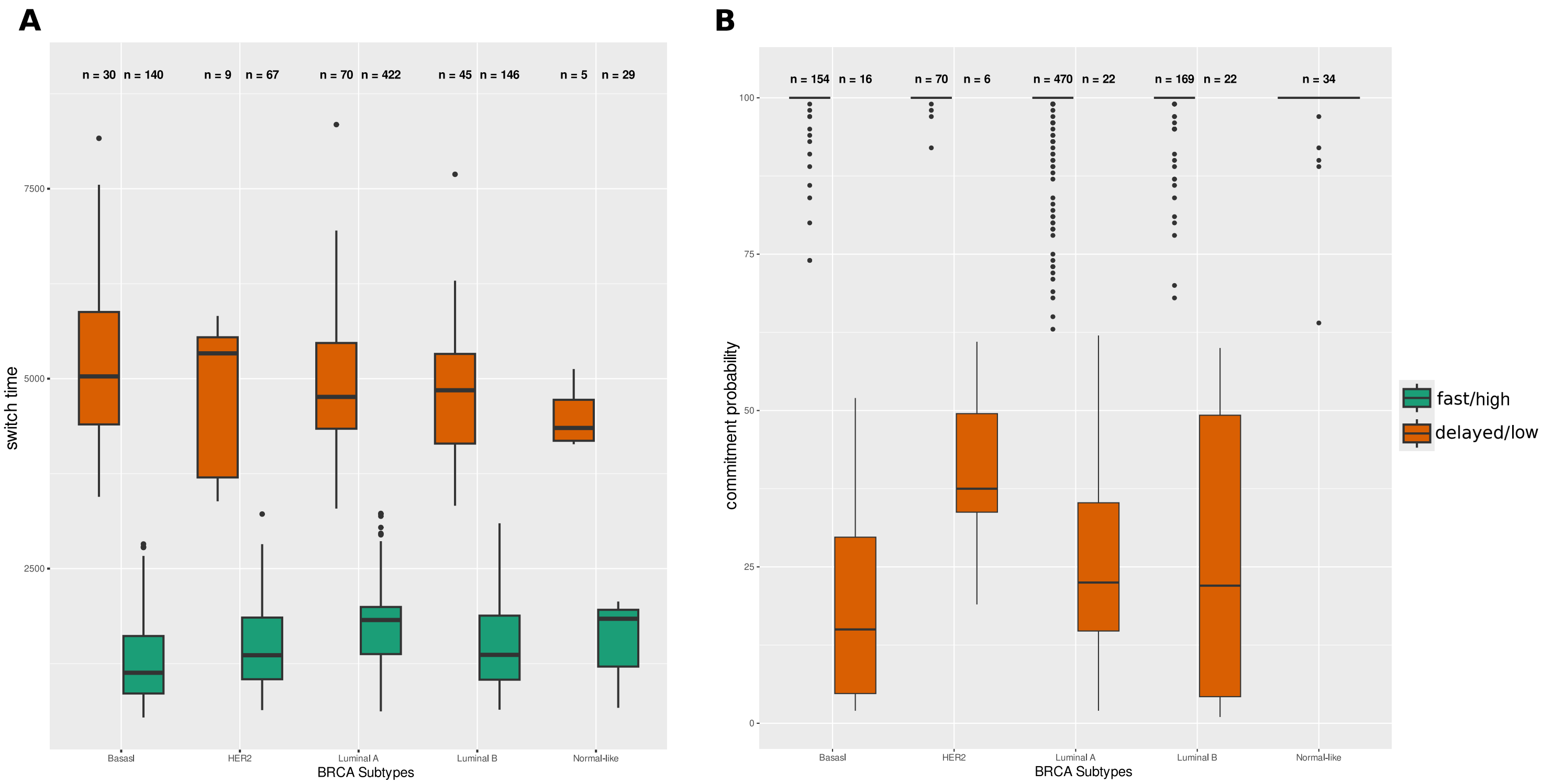

Supplementary Fig. 4. Distribution of patient metrics across subtypes, stratified by the two patient clustering groups. Figure A shows the mean switch time, where green indicates patients with a switch time clustered as 'fast', and orange indicates those clustered as 'delayed'. A similar analysis is presented for commitment probability (Figure B), where green represents patients with a high commitment to the G2/M transition, and orange represents those with low commitment based on the model.

8 Kaplan-Meier survival analysis across BRCA subtypes

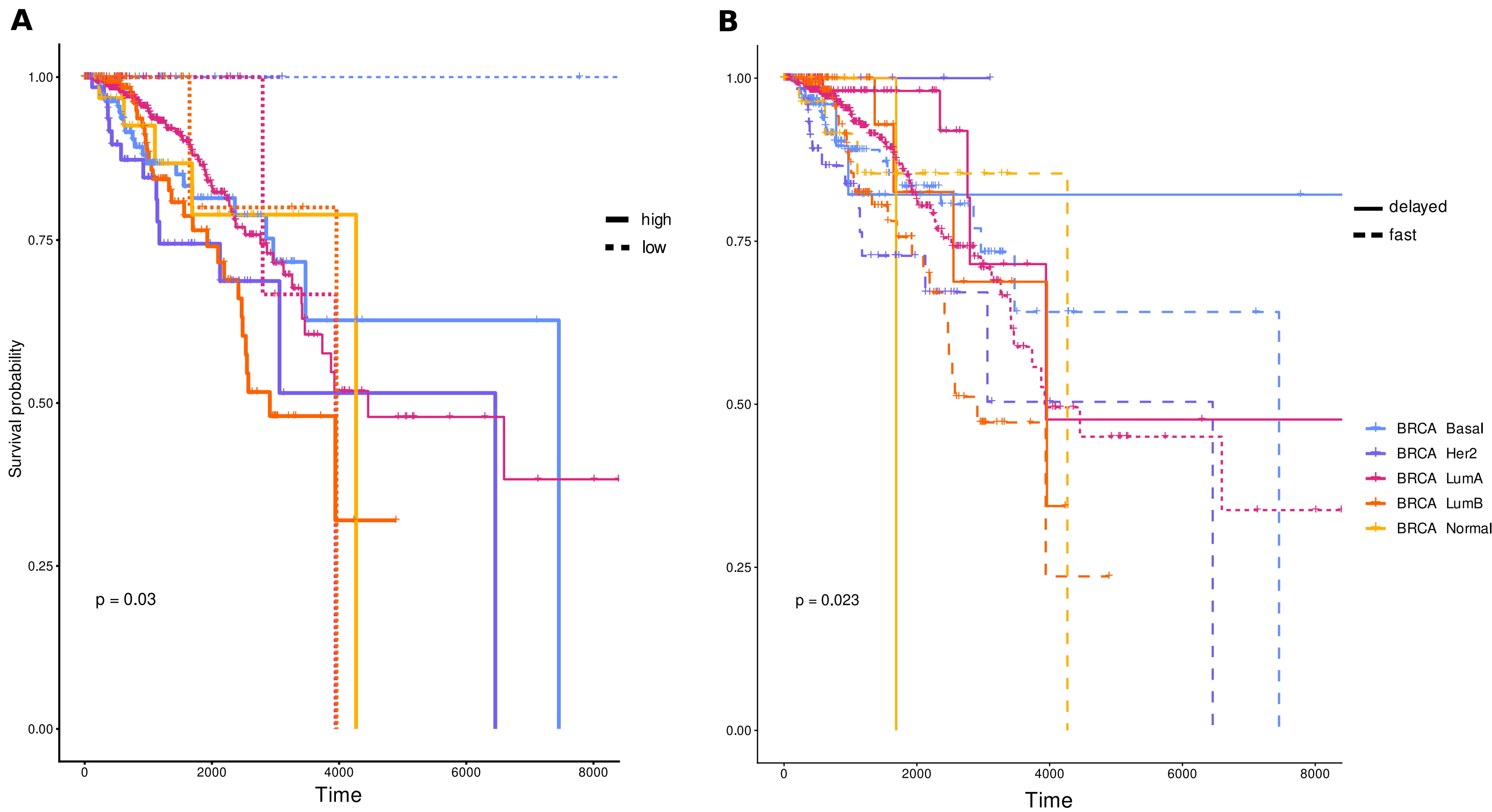

Supplementary Fig. 5. (A) Survival curves stratified by switch commitment percentage and subtype. (B) Survival curves stratified by mean switch time-derived dynamical regimes and BRCA subtype. These analyses show that the prognostic value of model-derived dynamical features for overall survival remains consistent across subtypes. While smaller subgroup sample sizes naturally reduce statistical power, these findings indicate that our model-derived stratification captures tumor dynamics beyond what is explained by molecular subtype alone.

9 Cox proportional hazards analysis adjusting for MKI67 expression

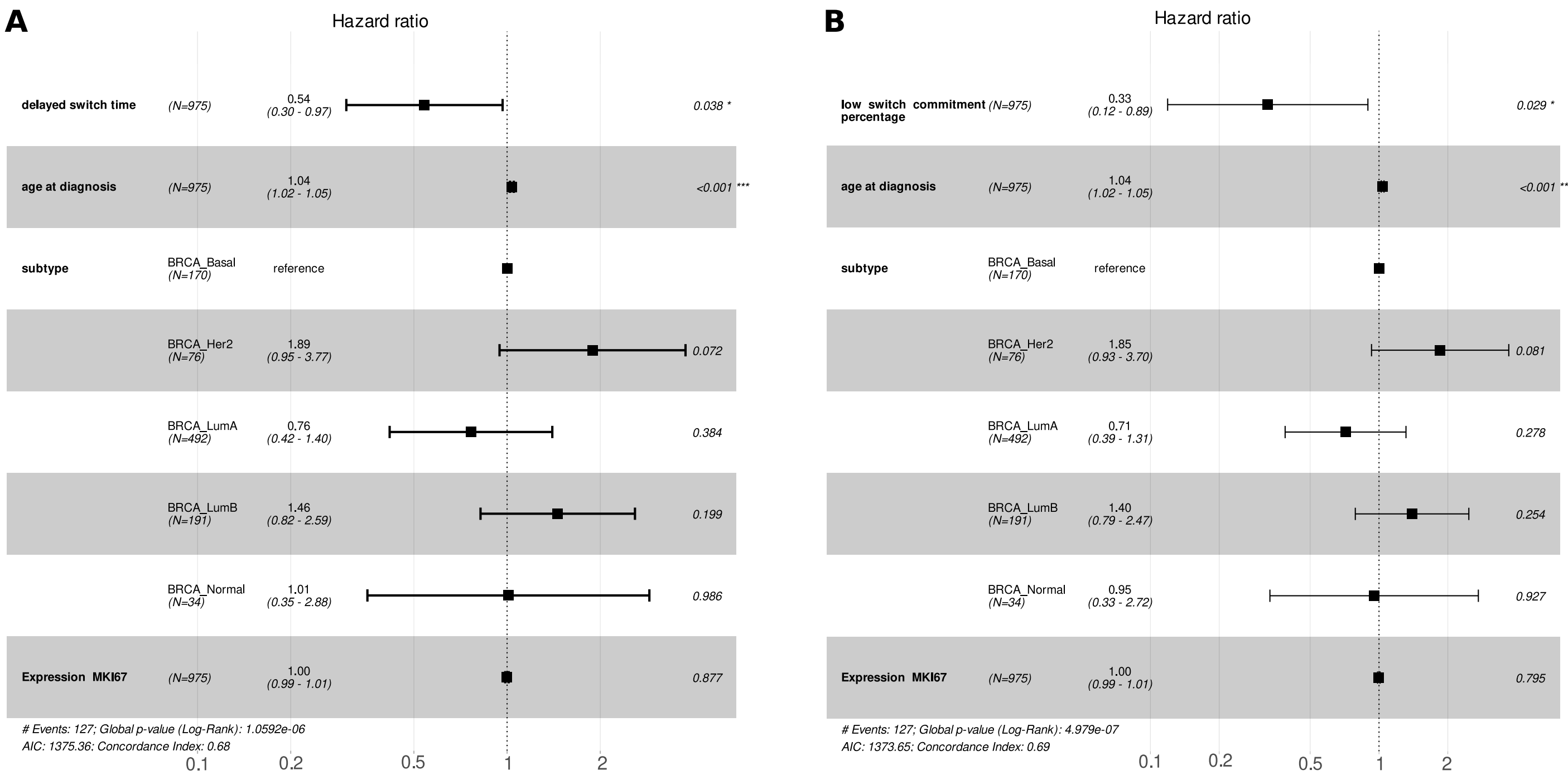

Supplementary Fig. 6. The associations between overall survival and model-derived dynamical features, delayed switch time (Figure A) and low switch commitment (Figure B),remained statistically significant after adjusting for MKI67, age, and molecular subtype. This suggests that these dynamical features reflect aspects of tumor behavior beyond those captured by standard proliferation markers alone.

10 Heatmaps of 21 OncotypeDx genes

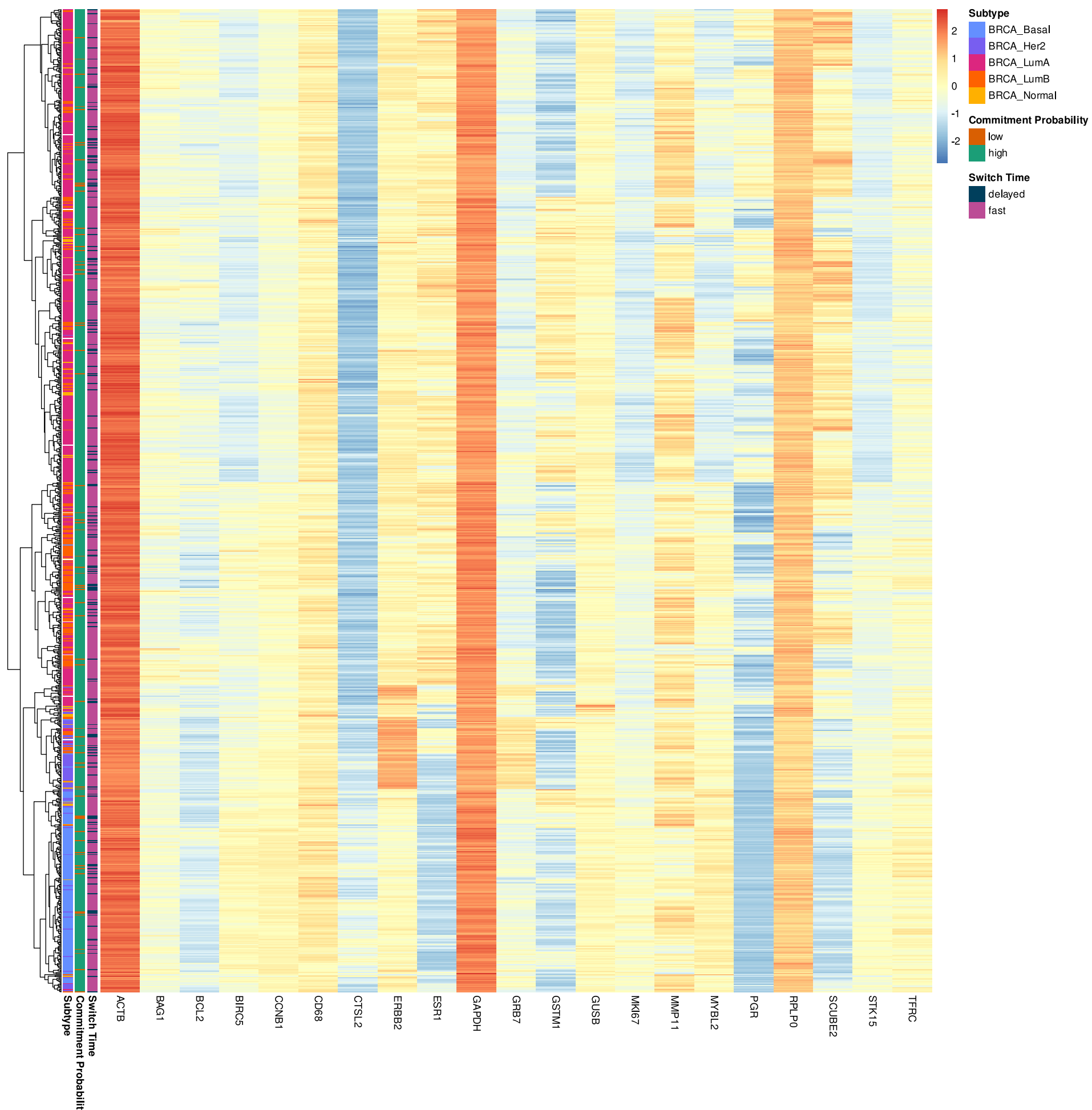

Supplementary Fig. 7. Hierarchical clustering of patients, utilizing Pearson correlation, was performed based on the expression of the 21 OncotypeDx genes. Heatmap annotated to highlight patient grouping based on commitment probability, switch time and BRCA subtypes. Notably, the samples did not clearly segregate according to the model-derived clusters, indicating that dynamical stratification is not merely a reflection of proliferation-associated transcriptomic profiles.

11 TCGA BRCA oncoplots for CNAs and point mutations

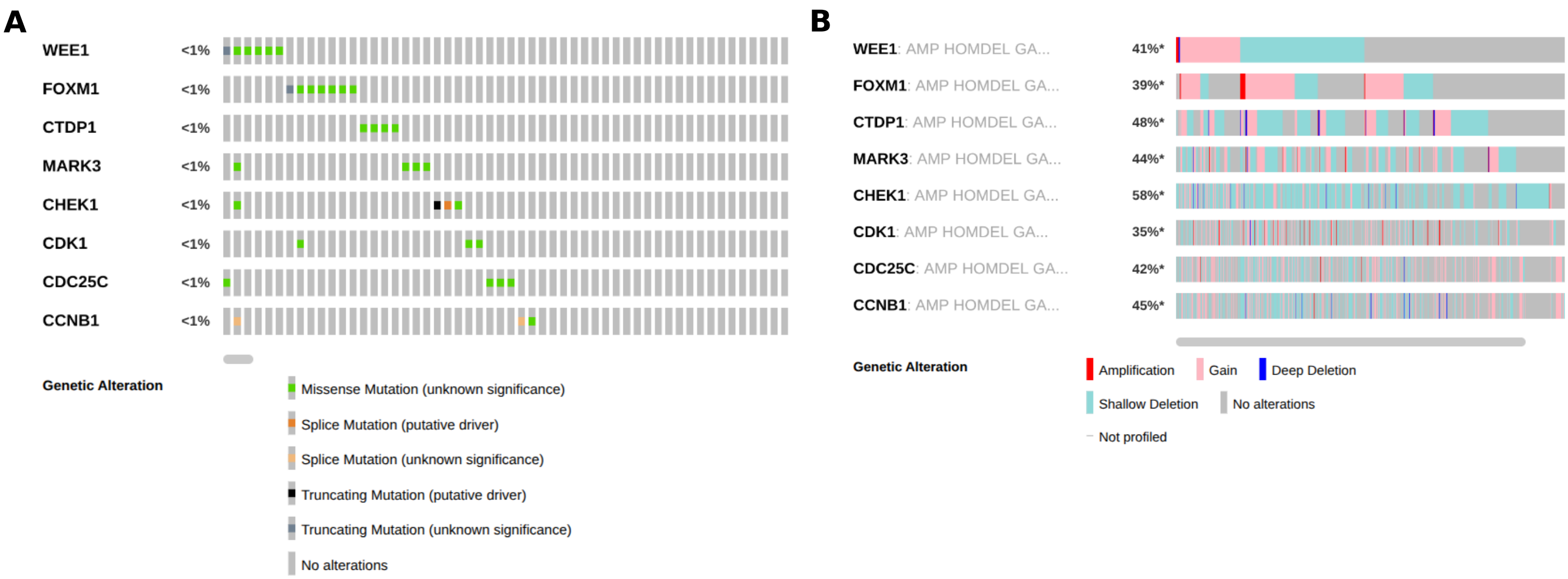

Supplementary Fig. 8. Oncoplots illustrating the frequency of (a) point mutations and (b) copy number alterations for the key components of the mathematical model. Protein nomenclature used in the model corresponds to the following genes: FCP1 (CTDP1), CHK1 (CHEK1), C-TAK1 (MARK3), and Cyclin B1 (CCNB1)

12 Comparison between Protein and mRNA expression

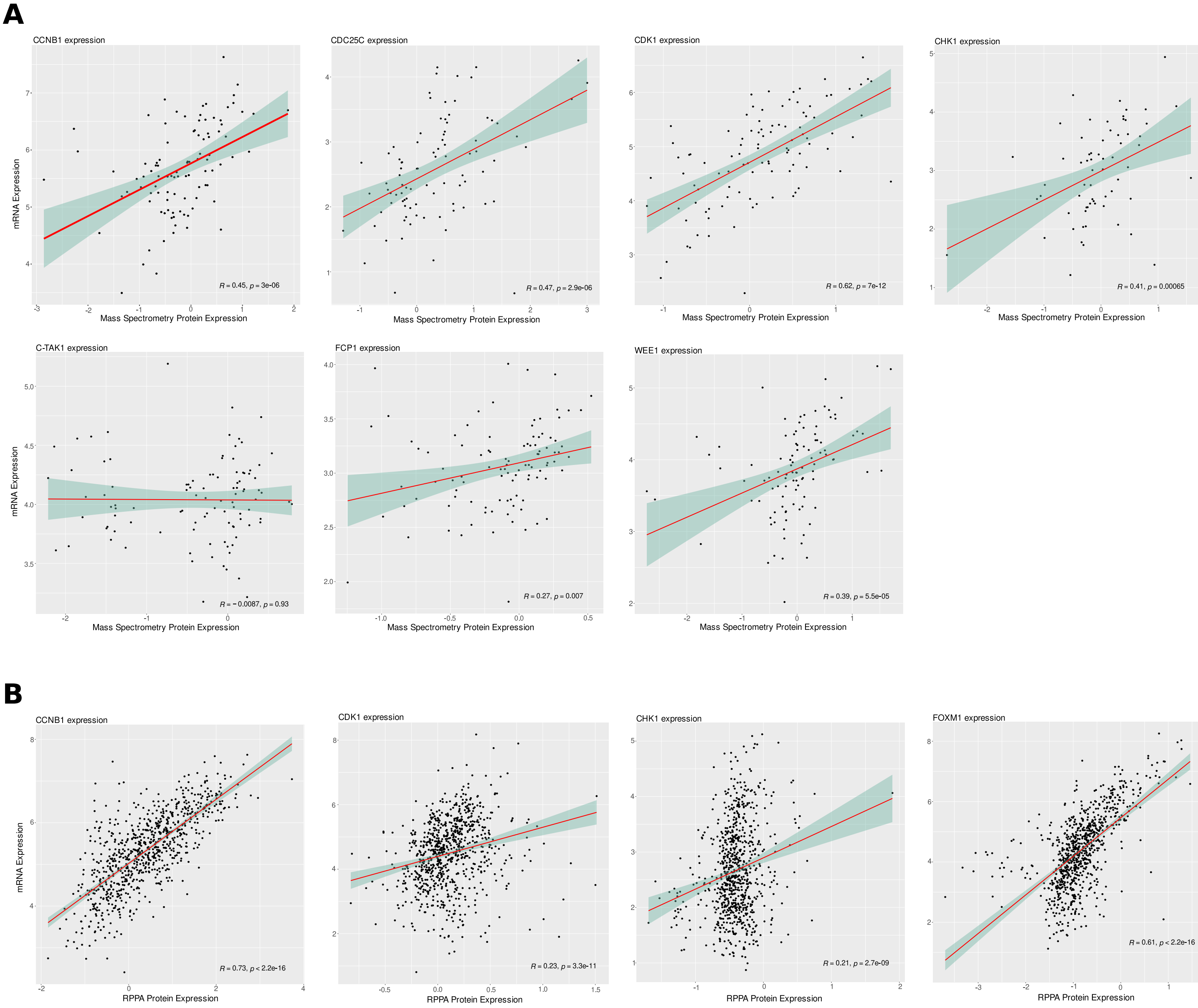

Supplementary Fig. 9. Relationship between log2-transformed mRNA and protein expression levels. From the cohort of 983 patients, protein data were available for 800 patients via RPPA (B) and 101 patients via Mass Spectrometry (A). Genes possessing both MS and RPPA data were analyzed using both modalities. A linear regression model is depicted by the red line, surrounded by a 95% confidence interval. Pearson correlation was also performed; the resulting correlation coefficient (R) and p-value are indicated on the plot.

13 Supplementary Data 1-4

Supplementary Data 1. Summary of TCGA BRCA cohort characteristics. Overview of clinical and genomic features of the TCGA breast cancer cohort analyzed in this study. The table reports the distribution of molecular subtypes, age at diagnosis and overall survival time (OS_time, in days). Summary statistics (median and interquartile range) are provided for continuous variables. In addition, the proportions of copy number alterations (deep deletion, shallow deletion, diploid, gain, amplification) are reported for the genes included in the G2/M model.

Supplementary Data 2. Clinical characteristics of TCGA BRCA cohort. Clinical and survival information for breast cancer patients from TCGA. The table includes patient ID, molecular subtype classification, age at diagnosis, overall survival status (OS; 0 = alive, 1 = deceased), and overall survival time (OS_time, in days).

Supplementary Data 3. Copy number variation status of cell cycle and checkpoint-related genes. Copy number alteration (CNA) status of model genes (FCP1, C-TAK1, FOXM1, CHK1, WEE1, and CDK1) across TCGA BRCA patients. CNA categories include Deep Deletion, Shallow Deletion, Diploid, Gain and Amplification.

Supplementary Data 4. Initial counts and parameters of patient-specific models. For each patient, initial counts for genes FCP1, C-TAK1, FOXM1, CHK1, and WEE1 are reported, together with the initial value of the parameter associated with CDK1.
